# E2F1‐activated SPIN1 promotes tumor growth via a MDM2‐p21‐E2F1 feedback loop in gastric cancer

**DOI:** 10.1002/1878-0261.12778

**Published:** 2020-08-26

**Authors:** Bei‐bei Lv, Ran‐ran Ma, Xu Chen, Guo‐hao Zhang, Lin Song, Su‐xia Wang, Ya‐wen Wang, Hai‐ting Liu, Peng Gao

**Affiliations:** ^1^ Key Laboratory for Experimental Teratology of Ministry of Education Department of Pathology School of Basic Medical Sciences Cheeloo College of Medicine Shandong University Jinan China; ^2^ Department of Pathology Shandong Provincial Hospital Affiliated to Shandong First Medical University Jinan China; ^3^ Department of Pathology Qilu Hospital Shandong University Jinan China; ^4^ Department of Pathology Yantai Yuhuangding Hospital of Qingdao University Yantai China; ^5^ Department of Breast Surgery, General Surgery Qilu Hospital of Shandong University Jinan China

**Keywords:** E2F1, gastric cancer, MDM2, SPIN1

## Abstract

Gastric cancer (GC) is one of the most common cancers around the world. Searching for specific gene expression changes during the development of GC could help identify potential therapy targets. We previously showed that the histone code reader SPIN1 may act as an oncogene in breast cancer. At present, the biological function and regulation of SPIN1 in GC remain unclear. Here, we demonstrate that SPIN1 is upregulated in GC tissues, compared with nontumorous gastric tissues. Increased expression of SPIN1 is closely associated with poor prognosis for patients with GC. Increased SPIN1 expression enhances GC cell proliferation, migration, and invasion and promotes cell cycle progression. Mechanically, SPIN1 sustains GC cell proliferation via activation of the MDM2‐p21‐E2F1 signaling pathway by binding to H3K4me3 of the MDM2 promoter region. Interestingly, E2F1 could directly bind to the SPIN1 promoter and activate its transcription, thus forming a positive feedback loop. Our data suggest that SPIN1 plays an important role in the development of GC and could be used as a promising prognostic biomarker and therapeutic target for GC.

## Graphical abstract

Gastric cancer (GC) is one of the most common cancers around the world. We demonstrate that SPIN1 is upregulated and associated with poor prognosis in GC. SPIN1 sustains GC cell proliferation via activation of MDM2‐p21‐E2F1 signaling pathway by binding to H3K4me3 of the MDM2 promoter. E2F1 could directly bind to the SPIN1 promoter and activate its transcription, forming a positive feedback loop.

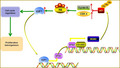

**Abbreviations**
Co‐IP: co‐immunoprecipitation
DFS: disease‐free survival
E2F1: adenovirus E2 factor 1
GC: gastric cancer
H3K4me3: trimethylation of lysine 4 on histone H3 protein subunit
LNM: lymph node metastasis
MDM2: murine double minute 2
OS: overall survival
P‐RB: phospho‐Rb
TCGA: The Cancer Genome Atlas

## 1 Introduction

Gastric cancer (GC) is one of the most common cancers, with the third highest lethality and fourth highest morbidity among all cancers worldwide [1]. Despite the considerable improvement in cancer diagnosis and comprehensive therapy, the 5‐year survival rate for GC remains relatively low due to tumor invasion and metastasis. Compared with other malignant tumors, such as lung cancer, breast cancer, and colorectal cancer, which have relatively effective molecular therapeutic targets, there is still no effective targeted therapy for GC. Searching for sensitive and specific genetic changes in the development of GC is important for the diagnosis and therapeutic purposes.

SPIN1, a member of the SPIN/SSTY family, was initially described as a major maternal transcript expressed in the unfertilized mouse egg during the transition from oocyte to embryo [2]. Recent studies have shown that human SPIN1 is upregulated in various types of malignant tumor tissues, including ovarian cancer, liposarcoma, breast cancer, and glioma, and may act as an oncogene that is implicated in tumorigenesis and progression [3, 4, 5, 6, 7]. Our previous investigation demonstrated that SPIN1 may act as an oncogene in breast cancer [4]. However, whether SPIN1 could promote the development of GC has not been established. Besides, no study on the transcriptional regulation mechanism of SPIN1 has been conducted to date, which could be important to understanding the oncogenic role of SPIN1. Furthermore, analysis of The Cancer Genome Atlas (TCGA) genome database indicates that SPIN1 expression is markedly elevated in human gastric tumor samples compared with normal tissues, indicating that SPIN1 may play an oncogenic role in GC progression.

In this study, we demonstrate that SPIN1, which is upregulated by the transcription factor adenovirus E2 factor 1 (E2F1), is overexpressed in GC samples and is associated with GC patient survival. Then, we show that SPIN1 promotes GC cell proliferation via activating the MDM2‐p21‐E2F1 signaling pathway by binding with H3K4me3. Interestingly, suppression of SPIN1 expression by ShRNA‐SPIN1 lentiviral vector decreases tumor xenograft growth *in vivo*. This study reveals the mechanism of SPIN1 in promoting GC tumor growth and provides evidence that SPIN1 is a novel prognostic biomarker and a promising therapeutic target in GC.

## 2 Materials and methods

### 2.1 Tissue samples

A total of 228 cases of paraffin‐embedded tissues (including 113 cases of primary GC, 56 cases of secondary lymph node metastasis (LNM) foci, and 59 cases of nontumorous gastric mucosa adjacent to the carcinoma tissues) were collected from patients within the age range of 34‐90 years and who underwent surgical resection at Qilu Hospital of Shandong University between 2007 and 2010. Our study was approved by the Institute's Research Ethics Committee of Shandong University. The experiments were undertaken with the understanding and written consent of each subject. The study methodologies conformed to the standards set by the Declaration of Helsinki. The World Health Organization (WHO) classification (2010) and UICC/AJCC TNM classification (8th edition) were followed in pathological classification and tumor stage definition. All of the samples were fixed in 40 g·L^−1^ formaldehyde and embedded in paraffin for histological evaluation and immunohistochemistry.

### 2.2 Immunohistochemistry

Paraffin‐embedded tissue sections were prepared and immunostained with antibodies against SPIN1 (1 : 150; Proteintech, Wuhan, China, 12105‐1‐AP). The streptavidin–peroxidase–biotin (SP) immunohistochemical method and the immunostaining results were performed as previously described [8, 9]. To quantify protein expression, both the intensity and extent of immunoreactivity were evaluated and scored. Staining intensity was scored as follows: 0 = negative, 1 = weak positive, 2 = intermediate positive, and 3 = strong positive. The scores of the extent of immunoreactivity were determined according to the percentage of GC cells that showed positive staining in each microscopic field of view (0–100%). A final score ranging from 5 to 270 with the median score of 100 was achieved by multiplying the scores for intensity and extent. Overall scores of 5–100 were classified into the low expression group, whereas 100–270 comprised the high expression group. For the negative control (NC), the primary antibody was replaced with PBS.

### 2.3 Cell lines

All five human GC cell lines, including MKN45 (poorly differentiated), BGC823 (poorly differentiated), SGC7901 (metastatic GC cell line), AGS, HGC27, the immortalized gastric cell (GES‐1), and human embryonic kidney cell line HEK293T, were purchased from the American Type Culture Collection (authenticated using STR profile analysis, Manassas, VA, USA) and Shanghai Cancer Institute (Shanghai, China). These cell lines were confirmed to be mycoplasma‐negative.

### 2.4 Cell culture and transient transfection

MKN45, BGC823, and SGC7901 cells were cultured in RPMI 1640 (Gibco, Grand Island, NY, USA) supplemented with 10% FBS. HEK293T cells were cultured in Dulbecco's modified Eagle medium (Gibco) supplemented with 10% FBS. The entire SPIN1 coding sequences were cloned into the expression plasmid pcDNA3.1 (+) (pcDNA3.1 (+)‐SPIN1), and the empty plasmid pcDNA3.1 (+) was used as a control. Plasmids were transfected with TurboFect transfection reagent (Thermo Scientific™, Waltham, MA, USA). SPIN1 small interfering RNA (siRNA‐SPIN1; RiboBio, Guangzhou, China) or the respective NCs were transfected using X‐treme GENE transfection reagent (Roche, Indianapolis, IN, USA) according to the manufacturer's instructions. Cotransfection of SPIN1 vector and TP53 small interfering RNA (siRNA‐TP53; RiboBio) or siRNA‐SPIN1 and TP53 vector (CH811472; Vigene Biosciences, Jinan, China) was also transfected with TurboFect transfection reagent.

### 2.5 RNA extraction and real‐time quantitative PCR

Total RNA was extracted from the GC cells grown in 12‐well plates using TRIzol^®^ reagent (Invitrogen, Carlsbad, CA, USA) according to the manufacturer's manual. Then, cDNA was synthesized from 1 μg of the total RNA with a ReverTra Ace qPCR RT kit (Toyobo, Osaka, Japan). Real‐time quantitative PCR was then performed using SYBR Green Real‐time PCR Master Mix (Roche Diagnostic GmbH, Mannheim, Germany) and an Applied Roche LightCycler^®^ 96 instrument. Relative expression was normalized to GAPDH expression, which yielded a $2^{‐\Delta C_{t}}$ value.

### 2.6 Western blotting

The following primary antibodies were used in this study: SPIN1 (1 : 1000; Proteintech, 12105‐1‐AP), MDM2 (1 : 500; Santa Cruz, Dallas, TX, USA, sc‐5304), CDK4 (1 : 1000; CST, Beverly, MA, USA, 12790), CyclinD1 (1 : 1000; CST, 2978), CDK2 (1 : 1000; CST), CDK6 (1 : 1000; CST), p21 (1 : 1000; Santa Cruz, sc‐24559), p27 (1 : 1000; CST, 3686), p53 (1 : 800; Santa Cruz, 47698), E2F1 (1 : 1000; Millipore, Billerica, MA, USA, 2970117), P‐Rb (1 : 1000; CST, 8516), and β‐actin (1 : 1000; OriGene, Rockville, MD, USA, sc‐47778). The protein bands were detected using a FluorChem Q machine (Cell Biosciences, Inc., Santa Clara, CA, USA). Independent experiments for western blotting were performed at least thrice.

### 2.7 Cell migration, invasion, and proliferation assays

Cell migration and invasion capabilities were detected using Transwell chambers (Corning, NY, USA) either uncoated or coated with Matrigel matrix (BD, Science, Sparks, MD, USA) as previously described [10].

Cell Titer 96 nonradioactive cell proliferation (MTS) (Promega BioSciences, Madison, WI, USA), Cell‐Light™ EdU cell proliferation detection (EdU) (RiboBio), and colony formation assays were performed to test the proliferation ability of the GC cells following the manufacturer's protocols. For the MTS assay, cell proliferation was measured at 24, 48, and 72 h after transfection. MTS (5 mg·mL^−1^) was added to the culture medium, followed by an incubation of 2 h, and absorbances were read at a wavelength of 490 nm. For the EdU assay, after EdU incubation, the cells were treated with an Apollo reaction cocktail, stained with DAPI, and visualized under a fluorescent microscope (Olympus, Tokyo, Japan). For the colony formation assay, 500 cells were seeded into six‐well plates. After incubation for 2 weeks, the colonies were fixed and stained, and then, formative clones were counted.

### 2.8 Flow cytometry analysis of cell apoptosis and cell cycle

To evaluate apoptosis, the GC cells were collected 48 h after transfection and double stained with Annexin V‐fluorescein isothiocyanate (FITC) and propidium iodide (PI; Beyotime, Shanghai, China) using an FITC Annexin V Apoptosis Detection kit (BestBio, Shanghai, China). For the cell cycle assay, GC cells were collected 72 h after transfection. These cells were fixed with 75% ethanol overnight and then stained with PI at 37 °C for 30 min. The harvested cells were then analyzed by flow cytometry (FACScan; BD Biosciences, San Jose, CA, USA) based on the protocol provided by manufacturer.

### 2.9 Identification of the proximal promoter region and transcriptional factors of SPIN1

The 2000‐bp transcription start site (TSS) upstream sequence of SPIN1 was extracted from the SPIN1 sequence, which was downloaded from the UCSC Genome Browser (http://genome.ucsc.edu/). The putative SPIN1 promoter regions (−2022/0, −1004/0, −508/0, −374/0, −253/0, −150/0, and −75/0) were PCR‐amplified from the genomic DNA of BGC823 cells, which were then inserted into the *Hind*III‐*Nhe*I sites upstream of the firefly luciferase in the pGL3‐Basic vector (Promega, Madison, WI, USA). The constructs were named based on the location of the promoter fragments relative to the TSS. The dual‐Luciferase reporter assay was performed to identify the proximal promoter region as previously described [11]. Then, the proximal promoter sequence of SPIN1 was submitted to the JASPAR program (http://jaspar.genereg.net/) to identify possible transcriptional factors. The full‐length cDNA sequence of SP1 and TFIID was PCR‐amplified from the cDNA of BGC823 cells and cloned into the pcDNA3.1 vector. CEBPβ, E2F1, and YY1 vectors were purchased from Vigene Biosciences.

### 2.10 ChIP assay

ChIP assays were performed using the EZ‐Magna ChIP Chromatin Immunoprecipitation Kit (Millipore) according to the instructions of the manufacturer. Briefly, the MKN45 and BGC823 cells were crosslinked with 1% formaldehyde for 10 min at room temperature. After incubation with anti‐E2F1 (Millipore; #2970117), anti‐H3K4me3 (CST, C42D8), or IgG antibody and protein A/G magnetic beads overnight, immunoprecipitation was performed. Proteinase K was used to digest proteins, and chromatin was extracted and then used in PCR and RT‐qPCR analyses.

### 2.11 Co‐immunoprecipitation assays

Briefly, 500–1000 μg of BGC823 proteins were incubated with anti‐SPIN1 at 4 °C overnight. Protein A/G beads (Santa Cruz Biotechnology) were then added, and the mixture was incubated at 4 °C for additional 1–2 h. The beads were washed five times with lysis buffer. The bound proteins were then detected using primary antibodies H3K4me3 (CST, C42D8) by western blot analysis.

### 2.12 Tumor xenograft model and ShRNA‐SPIN1 recombinant lentiviral vector treatment *in vivo*

The construction of the tumor xenografts model was conducted at the Laboratory Animal Center of Shandong University (Jinan, China). All animal experiments were carried out in accordance with the Guide for the Care and Utilization of Laboratory Animals (Shandong University) and were approved by the Committee on the Ethics of Animal Experiments of the Shandong University. Four‐week‐old male BALB/c nude mice (Huafukang Biotechnology, Beijing, China) were grown in a sterile room kept at a constant humidity and temperature (25‐28 °C) with a 12‐h light–dark cycle. After one week of acclimatization, the SGC7901 cells (density: 7 × 10^5^) were resuspended in 200 μL phosphate‐buffered saline and subcutaneously injected into the right lateral of axilla region. Ten days after injection (the tumors were then about 0.6‐1.0 cm in diameter), 14 mice were divided into two groups. Then, the same volume (0.1 mL) of the control vector (LV‐NC) and LV‐shRNA‐SPIN1 was injected into multiple sites intratumorally in two different groups every seven days (a total of three injections). The size of the tumors was measured with Vernier calipers every three days, and tumor volumes were calculated using the equation: *V* = (Tumor length × Width^2^)/2. On day 31 after injection, the mice were sacrificed, and the tumor nodules, lungs, and livers were excised for further analysis.

### 2.13 Statistical analysis

Statistical analysis was performed using graphpad prism 7 (GraphPad Software, Inc., San Diego, CA, USA). The correlation between SPIN1 expression and clinicopathological parameters was performed using the chi‐squared test. Significant differences were confirmed using Student's *t*‐test for two groups or one‐way ANOVA for three groups. Survival rates were calculated using the Kaplan–Meier method, and differences among survival curves were examined using a logrank test. Each experiment was repeated thrice. *P* < 0.05 was considered statistically significant.

## 3 Results

### 3.1 SPIN1 is upregulated in human GC tissues and is associated with poor prognosis

**Fig. 1:**
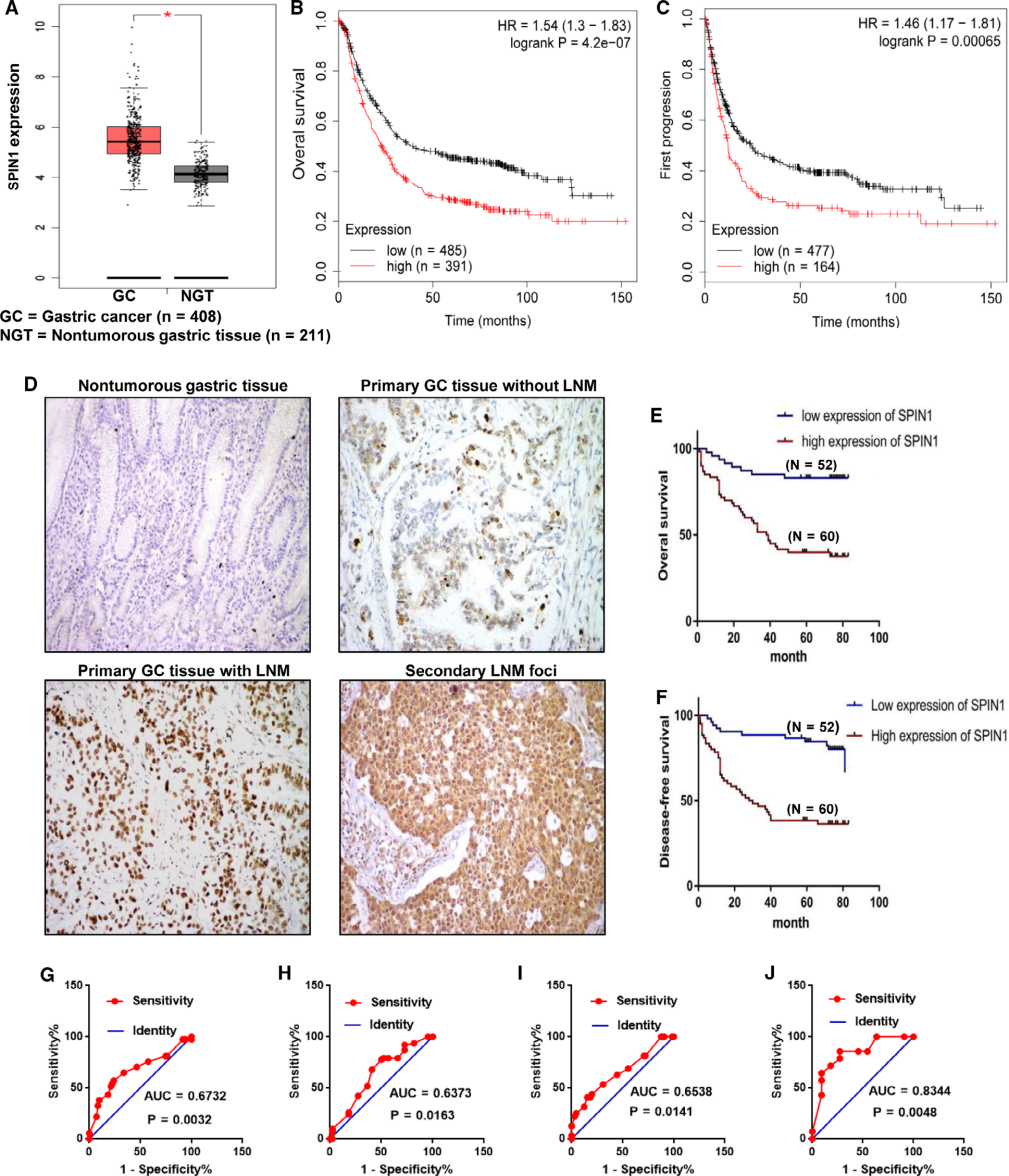
Identification and validation of SPIN1 as a tumor‐associated gene in GC. (A) SPIN1 expression is markedly elevated in human gastric cancer samples compared with normal tissues from the TCGA databases using GEPIA (*n* = 619, *t*‐test, *P* < 0.05). Data are presented as mean ± SD. (B, C) The Kaplan–Meier Plotter showed that patients with high expression of SPIN1 had poorer overall survival (Fig. 1B, *n* = 876, logrank *P* = 4.2e‐07) and first progression survival (Fig. 1C, *n* = 641, *P* = 0.00065) than those with low expression of SPIN1. (D) SPIN1 protein expression is confirmed immunohistochemically in GC paraffin‐embedded samples. SPIN1‐positive staining is predominantly localized in the nucleus. SPIN1 is negative in normal gastric mucosa, weak intensity with low positivity rate in primary gastric cancer tissues without LNM, strong intensity with high positivity rate in primary gastric cancer tissues with LNM and in secondary lymph node metastatic foci (magnification ×100). (E, F) Kaplan–Meier curves shows that the patients with higher SPIN1 expression have poorer overall survival (E, logrank test, *P* < 0.0001) and disease‐free survival (F, logrank test, *P* < 0.0001). (G–J) To test the ability of SPIN1 as a diagnostic marker for clinical pathological parameters related to gastric cancer, ROC curves were established. ROC curves reflect separation between GC with and without LNM (G), early and late clinical stage (H), high and low differentiation (I), with and without distant metastasis (J).

At first, we used GEPIA (http://gepia.cancer‐pku.cn/index.html) to analyze published data from TCGA for SPIN1 expression in GC. The results indicated that SPIN1 expression is markedly elevated in human gastric cancer samples compared with normal tissues (Fig. 1A, *n* = 619, *P* < 0.05). Moreover, survival analysis using the Kaplan–Meier Plotter showed that patients with high expression of SPIN1 had poorer overall survival (Fig. 1B, *n* = 876, logrank *P* = 4.2e‐07) and first progression survival (Fig. 1C, *n* = 641, *P* = 0.00065) than those with low expression of SPIN1.

By IHC assay, SPIN1‐positive staining was located mainly in the nuclei of cells in both GC and nontumorous tissues. SPIN1 expression exhibited a gradual increase from nontumorous gastric mucosa (8.47%, 5/59) via primary GC tissues (53.98%, 61/113) to secondary LNM foci (69.64%, 39/56) (Table S1, chi‐square test; Fig. 1A, *t*‐test; *P* < 0.05). More interestingly, we found that SPIN1 expression in GC with LNM was significantly higher than that without LNM (Fig. 1D), suggesting that SPIN1 may be associated with lymph node metastasis in GC.

To further assess the clinical significance of SPIN1 in GC, we correlated SPIN1 expression with clinical variates of GC cohorts. The results showed that high SPIN1 expression was strongly correlated with positive LNM (*P* = 0.0426), positive distant metastasis (*P* = 0.0250), clinical stage (*P* = 0.0011), and differentiation (*P* = 0.0172), whereas there was no correlation between SPIN1 expression and other characteristics such as age, sex, and tumor size (Table 1). Kaplan–Meier analysis showed that high SPIN1 expression was correlated with poorer OS and DFS (Fig. 1E,F). Then, univariate and multivariate analyses by the Cox proportional hazards regression model were performed to explore factors associated with patient outcome. The results indicated that SPIN1 expression, tumor stage, LNM, and distant metastasis were significantly correlated with OS of GC patients, whereas only distant metastasis was an independent prognostic predictor for OS (Table S2, *P* < 0.05). To test the ability of SPIN1 as a biomarker to distinguish different clinical pathological parameters related to GC patients, ROC curves were established. ROC curves reflected clear separation between GC with and without LNM (Fig. 1G), early and late clinical stage (Fig. 1H), high and low differentiation (Fig. 1I), and with and without distant metastasis (Fig. 1J). These results indicated that SPIN1 might play an important role in the pathogenesis of GC and could potentially be used as a prognostic biomarker for GC patients.

**Table 1:** Association between SPIN1 expression and clinicopathologic factors in 112 primary gastric cancer.

	Low expression of SPIN1	High expression of SPIN1	*P* value
Tumor size			
≤ 5.5 cm	30	30	0.6981
> 5.5 cm	21	25	
Miss	1	5	
Age (year)			
≤ 61	24	32	0.5700
> 61	28	28	
Gender			
Male	35	48	0.1369
Female	17	12	
Lymph node metastasis			
Yes	30	46	0.0426
No	22	14	
Clinical stage			
I, II	32	18	0.0011
III, IV	20	42	
Differentiation			
Moderately	25	15	0.0172
Poorly	27	45	
Distant metastasis			
Yes	5	17	0.025
No	47	43	

### 3.2 Identification of the proximal promoter regions of SPIN1

**Fig. 2:**
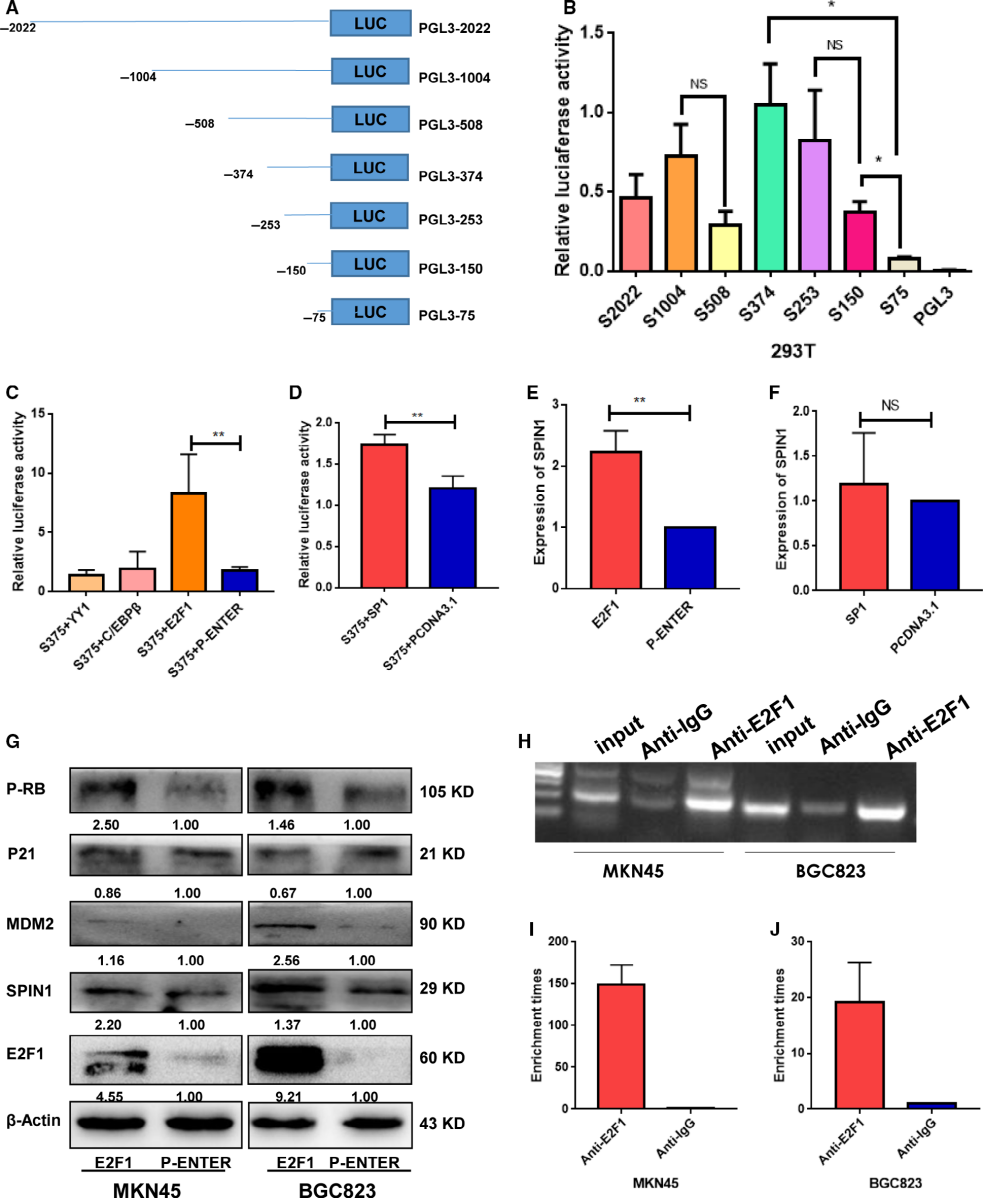
E2F1 promotes transcription of SPIN1. (A) Schematic diagram of the putative SPIN1 promoter regions (−2022/0, −1004/0, −508/0, −374/0, −253/0, −150/0, and −75/0). These promoter fragments were PCR‐amplified from the genomic DNA of BGC823 cells, which were then inserted into the HindIII‐NheI sites upstream of the firefly luciferase in the pGL3‐Basic vector. (B) Transcriptional activity analysis of the putative SPIN1 promoter fragments in 293T cells by dual‐luciferase reporter assays. Three independent experiments were performed, and data are presented as mean ± SD (unpaired *t*‐test with Welch's correction,*P < 0.05). (C, D) Dual‐luciferase reporter assays show that E2F1 and SP1 increase promoter activities of pGL3‐374/0. Three independent experiments were performed, and data are presented as mean ± SD (unpaired *t*‐test, **P < 0.01). (E, F) RT‐qPCR assay indicates that E2F1 promotes the expression level of SPIN1 in MKN45 cells, whereas SP1 does not. Three independent experiments were performed, and data are presented as mean ± SD (unpaired *t*‐test, **P < 0.01). (G) Western blot assay indicates that E2F1 promotes the expression level of SPIN1, as well as its target genes MDM2, P‐RB, while the expression level of p21 is decreased. Three independent experiments were performed. (H) ChIP‐PCR assay shows that PCR products were amplified from the DNA fragment which was immunoprecipitated by the anti‐E2F1 antibody using primers that covered the four E2F1‐binding sites. Three independent experiments were performed. (I, J) Approximate 150‐fold (MKN45) and 20‐fold (BGC823) enrichment of the promoter amplifications of SPIN1 in the four binding sites in GC cells was observed. Three independent experiments were performed, and data are presented as mean ± SD.

To explore the molecular mechanisms underlying SPIN1 upregulation, we performed promoter analysis to search for potential transcription factor. To identify the core promoter region of SPIN1, the promoter activities of the region ~ 2000 bp upstream of its TSS and seven deletion constructs (−2022/0, −1004/0, −508/0, −374/0, −253/0, −150/0, and −75/0) were determined using dual‐luciferase reporter assays (Fig. 2A). The pGL3‐2000/0 plasmid showed strong luciferase activity compared to the luciferase reporter vector pGL3‐Basic. No statistically significant difference in luciferase activity between pGL3‐2000/0 and the deletion constructs, including pGL3‐1004/0, pGL3‐508/0, and pGL3‐374/0, was observed, while further deletion of up to pGL3‐75/0 resulted in an obvious decrease in luciferase activity relative to that of pGL3‐374/0 and pGL3‐150/0 (Fig. 2B). These findings suggested that the region 374 −75 bp upstream of the TSS of SPIN1 is its proximal promoter.

### 3.3 SPIN1 is directly regulated by transcription factor E2F1

To comprehensively identify the transcription factors binding to the promoter region of SPIN1, the transcription factor‐binding site region from −374 to −75 was analyzed using the JASPER program and PROMO (http://alggen.lsi.upc.es.). There were potential binding sites for the transcription factor SP1, E2F1, YY1, and C/EBPβ in the proximal promoter region of SPIN1. To determine whether these potential transcription factors could regulate SPIN1 expression, SP1, E2F1, YY1, and C/EBPβ were overexpressed in MKN45 and BGC823 cells that were transiently transfected with the pGL3‐374/0 construct. The results showed that the promoter activities of pGL3‐374/0 significantly increased in the E2F1 and SP1 overexpression groups relative to the NC, but not in the YY1 and C/EBPβ groups (Fig. 2C,D). Next, RT‐qPCR and western blotting also showed that E2F1 promotes the expression of SPIN1 at the mRNA and protein levels (Fig. 2E–G), whereas SP1 does not. To corroborate this notion, a ChIP assay was performed to address whether E2F1 binds to the SPIN1 promoter region. The results revealed that PCR products were amplified from the DNA fragment that was immunoprecipitated by the anti‐E2F1 antibody using primers that covered the four E2F1‐binding sites (Fig. 2H). Additionally, an approximately 150‐fold (MKN45) and 20‐fold (BGC823) enrichment of the promoter amplifications of SPIN1 in the four E2F1‐binding sites were observed using the anti‐E2F1 antibody (Fig. 2I,J). Taken together, these data demonstrate that E2F1 regulates the transcription of SPIN1 by directly binding to its promoter.

### 3.4 SPIN1 enhances GC cell migration, invasion, and proliferation

**Fig. 3:**
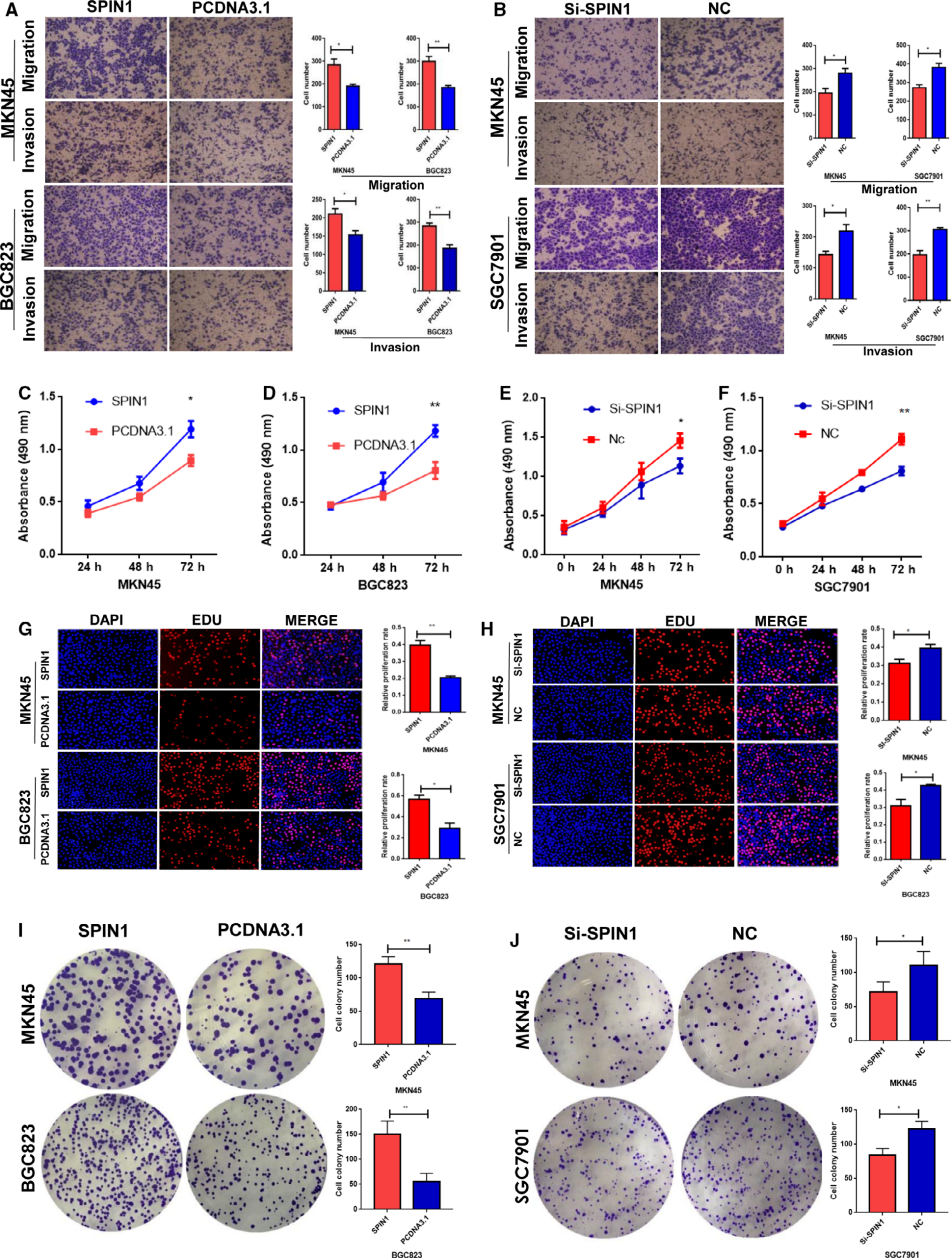
SPIN1 promotes GC cells migration, invasion, and cell proliferation *in vitro*. (A, B) Migration and invasion ability of the MKN45 and BGC823 cell lines by Transwell assays (magnification ×200). Three independent experiments were performed, and data are presented as mean ± SD (unpaired *t*‐test, *P < 0.05, **P < 0.01). (C–F) Cell proliferation rates were measured using MTS assay in MKN45, BGC823 and SGC7901 cells. Each experiment was performed in triplicate, and data are presented as mean ± SD. (G, H) EdU assay confirmed that ectopic expression of SPIN1 increased GC cell proliferation, whereas inhibition of SPIN1 expression significantly reduced their proliferation (magnification ×100). Three independent experiments were performed, and data are presented as mean ± SD (unpaired *t*‐test, *P < 0.05, **P < 0.01). (I, J) Colony formation assays showed that overexpression of SPIN1 increases colony formation capacity, whereas inhibition of SPIN1 expression reduces colony formation capacity (no magnification). Three independent experiments were performed, and data are presented as mean ± SD (unpaired *t*‐test, *P < 0.05, **P < 0.01).

We first verified the expression of SPIN1 in five GC cell lines using RT‐qPCR. The results showed that the expression of SPIN1 was upregulated in SGC 7901, which is a poorly differentiated and metastatic cell line, compared with MKN45 and BGC823 (Fig. S1A). RT‐qPCR analysis showed that the mRNA expression of SPIN1 was significantly up‐ or downregulated when transfected with SPIN1 plasmid or SiRNA‐SPIN1 in GC cell lines (Fig. S1B,C). Migration and invasion assays showed that the cells transfected with the SPIN1 plasmid exhibited enhanced migration and invasion activities compared with the control, whereas the cells in which SPIN1 was knocked down had decreased migratory and invasive capacities (Fig. 3A,B).

To explore the role of SPIN1 on cell proliferation, MTS, EdU, and colony formation assays were performed. The MTS assays suggested that SPIN1 overexpression significantly enhances GC cell growth, whereas SPIN1 knockdown inhibits their growth (Fig. 3C–F). The EdU assays confirmed that ectopic expression of SPIN1 increased GC cell proliferation, whereas inhibition of SPIN1 expression significantly reduced their proliferation (Fig. 3G,H). In addition, colony formation assays indicated that overexpression of SPIN1 increases colony formation capacity, whereas inhibition of SPIN1 expression reduces colony formation capacity (Fig. 3I,J). Together, these results suggest that SPIN1 enhances GC cell migration, invasion, and proliferation.

### 3.5 SPIN1 promotes GC cell cycle progression and has no effect on apoptosis of GC cells

**Fig. 4:**
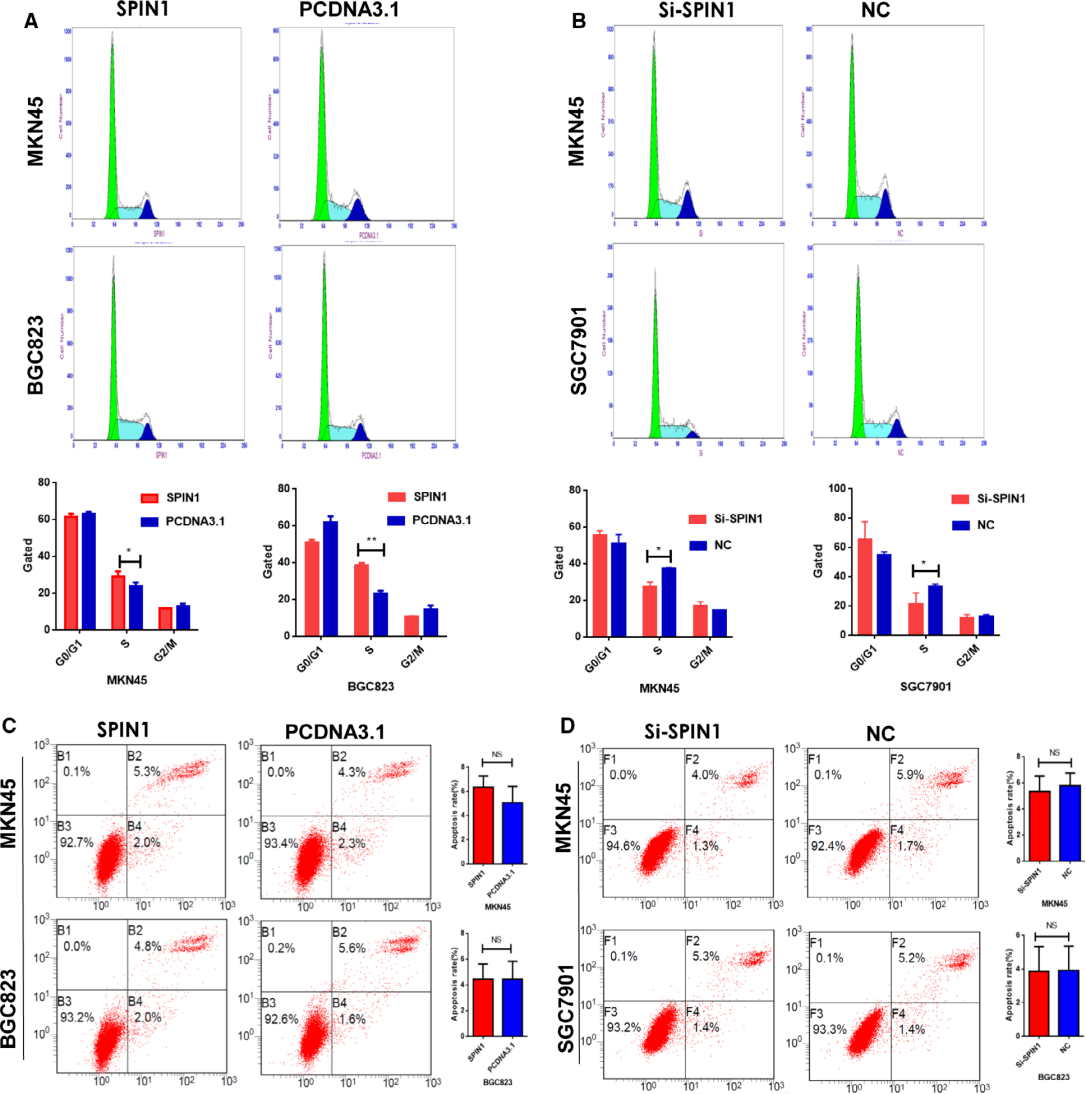
The effect of SPIN1 on gastric cancer cell cycle and apoptosis *in vitro*. (A, B) The cell cycle results indicated that SPIN1 overexpression promotes the transition from G1 to S phases of the cell cycle, whereas suppression of SPIN1 induced a decrease in the number of cells in the S phase and an unstable number of cells in the G2 phase. Three independent experiments were performed, and data are presented as mean ± SD (unpaired *t*‐test, *P < 0.05, **P < 0.01). (C, D) Annexin V‐FITC assays indicated that there was no difference in the proportion of apoptotic cells between the experimental and control groups. Three independent experiments were performed, and data are presented as mean ± SD (unpaired *t*‐test).

As SPIN1 can promote GC cell proliferation, we next determined whether SPIN1 overexpression or knockdown could affect the cell cycle or apoptosis in GC cells. The results showed that SPIN1 overexpression promotes the transition from G1 to S phases of the cell cycle (Fig. 4A), whereas suppression of SPIN1 induces a decrease in the number of cells in the S phase and an unstable number of cells in the G2 phase (Fig. 4B). These results showed that SPIN1 plays a pivotal role in the G1/S phase transition.

Next, we investigated the effects of SPIN1 on cell apoptosis. Annexin V‐FITC assays indicated that there was no difference in the proportion of apoptotic cells between the experimental and control groups (Fig. 4C,D). These results revealed that SPIN1‐mediated promotion of GC cell proliferation was due to cell cycle modulation rather than apoptosis.

### 3.6 SPIN1 promotes tumorigenesis and proliferation via the activation of MDM2‐p21‐E2F1 pathway

To identify the mechanism underlying the activation of GC cell proliferation by SPIN1, gene expression analysis was performed in our previous study (KangChen Inc., Shanghai, China, Project Code: H1408026) [4]. Based on considering the differentially expressed genes with an absolute fold‐change > 3, *P* < 0.05, and reported previously as known oncogenes with high expression in various tumors, including GC [12, 13, 14, 15, 16], four cancer‐associated genes including MAP2, MDM2, MAPKBP1, and GPNMB were selected as candidates to be investigated. Then, we validated the candidates by RT‐qPCR and western blotting. The results showed that the mRNA levels and protein expression of MDM2 increased when SPIN1 was overexpressed, while no significant effects on mRNA and protein expression of MAP2, MAPKBP1, and GPNMB were observed (Fig. S1G,H). Hence, we chose MDM2 for further investigation.

**Fig. 5:**
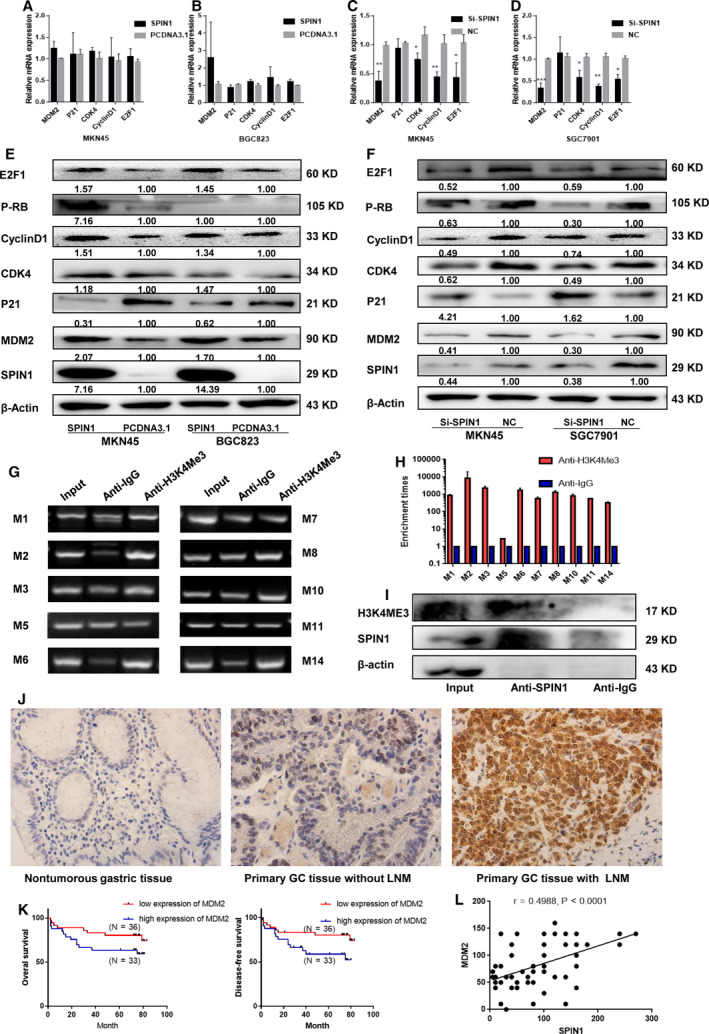
SPIN1 promotes tumorigenesis and proliferation via activation of MDM2‐p21‐E2F1 pathway through binding with H3K4me3. (A–D) RT‐qPCR revealed that the mRNA levels of MDM2, CDK4, CyclinD1, and E2F1 were suppressed by SPIN1 siRNA, while little effect on the mRNA expression was found when SPIN1 was overexpressed. Three independent experiments were performed, and data are presented as mean ± SD. (E, F) Western blot analysis verified that the protein levels of MDM2, CDK4, CyclinD1, P‐RB, and E2F1 were increased, and p21 was decreased after SPIN1 overexpressed in MKN45 and BGC823 cells, while SPIN1 knockdown exhibited the opposite result. Three independent experiments were performed. (G) CHIP‐PCR assay showed that H3K4me3 directly interacted with the H3K4me3 binding sites within MDM2 promoter in MKN45 cells. M1, M2, M3, M5, M6, M7, M8, M10, M11, and M14 represent primers that covered the H3K4me3 binding sites respectively. (H) CHIP‐qPCR analysis indicated higher fold enrichment of promoter amplifications of H3K4me3 in the anti‐H3K4me3 group than that of the anti‐IgG group in MKN‐45 cells. Three independent experiments were performed, and data are presented as mean ± SD. (I) Co‐IP assay validated the interaction between endogenous SPIN1 and H3K4me3 in MKN45 cells using anti‐SPIN1 antibody. Three independent experiments were performed. (J) MDM2 was negative in nontumorous tissues (*n* = 33), and expression of MDM2 in GC with LNM (*n* = 33) was significantly higher than that without LNM (*n* = 36) (magnification ×400). (K) Kaplan–Meier analysis showed that high MDM2 expression was correlated with poorer OS and DFS. (L) The results of Spearman's correlation analysis showed a positive relationship between MDM2 and SPIN1.

As we all know, MDM2 is a crucial mediator of cell cycle regulation, we hypothesized that SPIN1 promotes cell cycle progression in GC through MDM2‐mediated signaling pathway. The expression of the MDM2, p21, p27, and cell cycle regulators CDK2, CDK4, CDK6, CyclinD1, phospho‐Rb (P‐RB), and E2F1 was examined by RT‐qPCR and western blotting. The results revealed that the mRNA levels of MDM2, CDK4, CyclinD1, and E2F1 were suppressed by SPIN1 siRNA, while little effect on the mRNA expression was found when SPIN1 was overexpressed (Fig. 5A–D). The protein expression of MDM2, CDK4, CyclinD1, P‐RB, and E2F1 increased, whereas p21 expression decreased when SPIN1 was overexpressed in both GC cell lines. Conversely, knockdown of SPIN1 decreased the protein expression levels of MDM2 and its targets, CDK4, CyclinD1, P‐RB, and E2F1, whereas p21 exhibited the opposite results (Fig. 5E,F). No significant effects in protein expression of p27, CDK2, and CDK6 were observed (Fig. S1D,E). Additionally, apoptosis‐related proteins BCL2 and BAD were also examined by western blotting. Consistently with cell apoptosis results, no significant effects in protein expression of BCL2 and BAD were observed (Fig. S1D,E).

To investigate the relevance of SPIN1 and MDM2 in GC tissues, MDM2 expression level was quantified with immunohistochemistry in both GC and nontumorous tissues. As expected, the results showed that MDM2 was negative in nontumorous tissues (*n* = 33), the expression of MDM2 in GC with LNM (*n* = 33) was significantly higher than that without LNM (*n* = 36) (Fig. 5J). Then, Kaplan–Meier analysis showed that high MDM2 expression was related to poorer OS and DFS (Fig. 5K). Results of Spearman's correlation analysis showed a positive relationship between MDM2 and SPIN1 (Fig. 5L). Next, to validate our results, we used GEPIA to analyze published data from TCGA for MDM2 expression in GC. Consistently, the results indicated that MDM2 expression is markedly elevated in human GC samples (*n* = 408) compared with normal tissues (*n* = 211) (Fig. S2A). Moreover, survival analysis using the Kaplan–Meier Plotter showed that patients with high expression of MDM2 had poorer overall survival (Fig. S2B). These data further demonstrated that SPIN1‐induced proliferation of GC was mediated via MDM2.

**Fig. 6:**
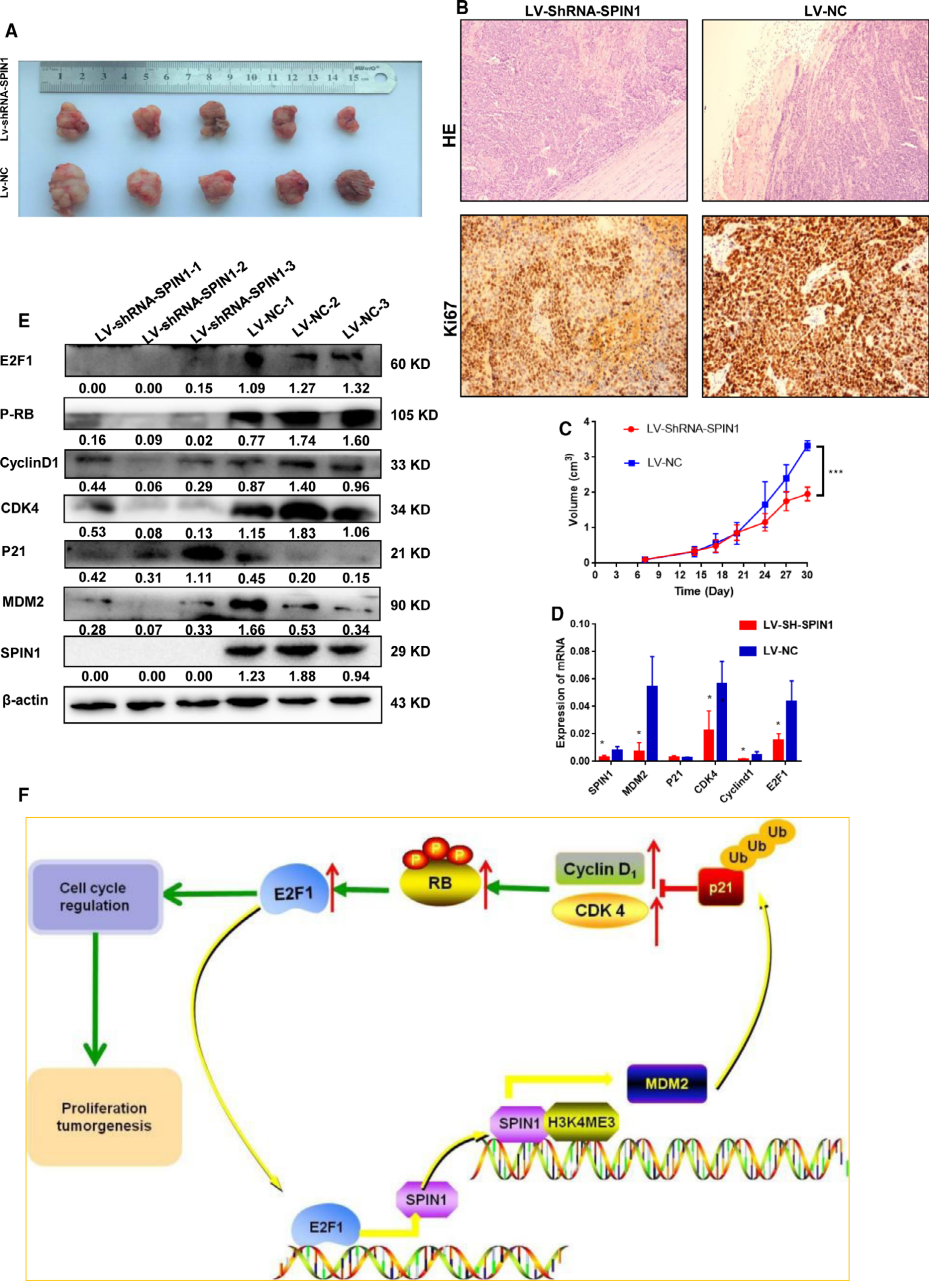
SPIN1 promotes tumorigenesis and proliferation via activation of MDM2‐p21‐E2F1 pathway *in vivo*. (A) After 3 times injection of LV‐NC (*n* = 5, 0.1 mL per mouse) and LV‐ShRNA‐SPIN1 (*n* = 5, 0.1 mL per mouse), on day 30, photographs of representative tumors are shown. (B) HE staining results showed that the tumors of LV‐Sh‐SPIN1 group (*n* = 5) were well‐encapsulated while LV‐NC group (*n* = 5) displayed local invasion into the muscle tissues (magnification ×100). Immunohistochemistry for Ki‐67 revealed that the GC cells in the LV‐shRNA‐SPIN1 group (*n* = 5) had a lower positivity rate than those in the LV‐NC group (*n* = 5) (magnification ×100). (C) Tumor size was measured using vernier caliper, and tumor volume was determined as described in Materials and methods section for 30 days. Each data point is the mean value of primary tumors (*n* = 5). Data are presented as mean ± SD. (D) RNA from fresh tumor tissues was extracted. qPCR showed that MDM2, CDK4, CyclinD1, and E2F1 mRNA expression were suppressed in the LV‐ShRNA‐SPIN1 group, but there was no significant difference in the p21 mRNA expression between two groups. Three independent experiments were performed, and data are presented as mean ± SD. (E) Western blotting confirmed the result that MDM2, CDK4, CyclinD1, P‐RB, and E2F1 protein expression were decreased in LV‐ShRNA‐SPIN1 group, while p21 was increased. Three independent experiments were performed. (F) Working model of the upstream regulatory and function mechanisms of SPIN1 in GC progression.

To further study the mechanism by which SPIN1 promotes GC progression *in vivo*, RNA and protein from fresh mice xenograft tumors were extracted. RT‐qPCR analysis showed that MDM2, CDK4, CyclinD1, and E2F1 mRNA expression were suppressed in the LV‐ShRNA‐SPIN1 group. However, there was no significant difference in p21 mRNA expression between the two groups (Fig. 6D). Western blotting confirmed the result that MDM2, CDK4, CyclinD1, P‐RB, and E2F1 protein expression decreased, whereas p21 expression increased in the LV‐ShRNA‐SPIN1 group (Fig. 6E). Taken together, these results revealed that SPIN1 promotes the cell cycle via the MDM2‐p21‐E2F1 signaling pathway in GC cells. Furthermore, E2F1 could in turn directly bind to the SPIN1 promoter region and activate its transcription (Fig. 2E–G), thus forming a positive feedback loop that drives the malignant behavior of GC.

### 3.7 SPIN1 enhances the expression of MDM2 by binding with H3K4me3 independently of p53

To further investigate the mechanism by which SPIN1 regulates the expression of MDM2, the UCSC Genome Browser (http://genome.ucsc.edu/) was used to predict upstream regulation of MDM2. H3K4me1, H3K4me3, and H3K27AC marks were found in seven cell lines from ENCODE (Fig. S1F). Previous and recent studies showed that SPIN1 is a histone code reader that can recognize H3K4me3 [17, 18, 19, 20]; therefore, we hypothesized that the methyl mark reader function of SPIN1 could confer a regulatory role in MDM2 expression. Then, CHIP‐qPCR assay was performed using the anti‐H3K4me3 antibody. The results revealed the existence of H3K4me3 in the MDM2 promoter region (Fig. 5G,H). Next, a Co‐IP assay was performed to validate the interaction between endogenous SPIN1 and H3K4me3 in GC cells using an anti‐SPIN1 antibody. The results also confirmed that SPIN1 directly binds to H3H4me3 (Fig. 5I). Taken together, these findings demonstrate that SPIN1 enhances the expression of MDM2 by binding with H3K4me3 of the MDM2 promoter.

MDM2 is an important target gene for p53. In line to establish whether the identified SPIN1‐MDM2‐p21‐E2F1 axis is p53‐dependent or not, we performed rescue experiments. At first, we used IARC TP53 database (https://p53.iarc.fr/CellLines.aspx) to specify the status of TP53 in three gastric cancer cell lines. The results indicated that MKN45 and BGC823 are wild‐type TP53 cell lines, while SGC7901 is mutant (Fig. S2C). Then, MKN45 and BGC 823 cells were cotransfected with SPIN1 vector and siRNA‐TP53, SGC7901 cell was cotransfected with si‐SPIN1 and wild‐type TP53 vector. The results of western blotting showed that when p53 is knockdown, SPIN1 overexpression still increased the protein expression of MDM2 in MKN45 and BGC823 (wild‐type TP53) cells (Fig. S2D). Likewise, when wild‐type TP53 is overexpressed, SPIN1 knockdown still decreased the protein expression of MDM2 in SGC7901 (mutant TP53) cell (Fig. S2E). These results indicated that regulation of MDM2 by SPIN1 is independent of P53.

### 3.8 SPIN1 could be used as a therapy target in suppressing GC growth *in vivo*

To explore whether SPIN1 can be used as a target for GC therapy, lentiviral packaged ShRNA‐SPIN1 was injected into the tumor xenografts model every seven days thrice. After a 30‐day follow‐up period, we found a significant reduction in tumor volume in the LV‐ShRNA‐SPIN1 groups (*n* = 5) compared with the LV‐NC group (*n* = 5) (Fig. 6A,C, *P* < 0.001). Besides, tumor nodules in the LV‐ShRNA‐SPIN1 group were noninvasive or well‐encapsulated. However, those tumors in the NC group showed invasion of the margin and into the surrounding stroma or muscle tissues (Fig. 6B). Immunohistochemistry for Ki‐67 revealed that the GC cells in the LV‐shRNA‐SPIN1 group had a lower positivity rate than those in the LV‐NC group (Fig. 6B). Taken together, these findings suggest that SPIN1 could be used as a target for GC therapy.

Based on these data and the aforementioned results, we hereby propose a model for the upstream regulatory and function mechanisms of SPIN1 in GC progression (Fig. 6F).

## 4 Discussion

Gastric cancer continues to be a significant health burden. The rates of GC and death from GC remain high in China. Extensive studies have been conducted to describe somatic genetic alterations in GC, with the intended goal of defining core biological pathways to facilitate the discovery of available targets for diagnostic and therapeutic purposes. Identification of tumor‐related genes might be critical for current effective and specific therapies for GC. SPIN1 is a histone code reader composed of three tudor‐like domains binding histone H3 trimethylated at lysine 4 (H3K4me3) with high affinity [18, 20]. As a transcriptional coactivator, SPIN1 has been reported to regulate the expression of ribosomal RNA (rRNA) [18]. It appeared to be a target for cell cycle‐dependent phosphorylation and was demonstrated to disrupt the cell cycle [21]. To our knowledge, no previous investigations have included a study of the functional roles and molecular mechanisms of SPIN1 in GC. Besides, no studies on the regulation mechanism of the upstream transcription factor and promoter analysis of SPIN1 have been reported to date.

In this study, we first found that SPIN1 is upregulated in GC tissues compared with nontumorous gastric tissues. Besides, patients with high levels of SPIN1 presented with worse OS and DFS, suggesting that SPIN1 functions as an important independent biomarker that could predict the clinical outcome of GC. Functional studies showed that SPIN1 can promote migration and proliferation of GC cells and also promote cell cycle progression. These results indicated that SPIN1 acts as a potential oncogene in GC. To further investigate the mechanism of high expression of SPIN1 in GC, we defined the proximal promoter of SPIN1 and confirmed E2F1 as a transcription factor to upregulate SPIN1 in GC. To our knowledge, the mechanism by which SPIN1 is upregulated in cancer remains unclear. E2F is a family of transcription factor proteins that has a variety of functions, including control of the cell cycle, cell differentiation, DNA damage response, and cell death. E2F1, a key regulator for the G1/S phase transition in the E2F family, was found to be dysregulated in a variety of cancers, including GC [22, 23, 24]. Our findings provide new insights into the transcriptional regulation of SPIN1.

Previous studies have shown that SPIN1 functions as an oncogene by activating the PI3K/AKT, WNT, and RET pathways [3, 4, 25, 26]. Given the importance of SPIN1 in the regulation of cell cycle [21], our results confirmed that SPIN1 initiates cell cycle progression and promotes cell proliferation in GC. Then, we found that SPIN1 promotes cell proliferation via the activation of the MDM2‐p21‐E2F1 pathway *in vitro* and *in vivo*. MDM2, a nuclear‐localized E3 ubiquitin ligase, is frequently overexpressed in human malignancies, making MDM2 an attractive therapeutic target [27, 28]. p21 is an important member of the family of cyclin‐dependent kinase inhibitors that has been recently discovered. Its function in the cell cycle arrest is largely dependent on p21 post‐translational modification [29]. Jin *et al*. [30] found that MDM2 directly inhibits p21 function by reducing p21 stability in a ubiquitin‐independent fashion. Based on the results of gene expression analysis, we verified that ectopic SPIN1 and knockdown of SPIN1 increased and decreased the expression of MDM2. Also, we validated that the downstream genes of MDM2 such as p21, CDK4, CyclinD1, P‐RB, and E2F1 were regulated by SPIN1. These results suggest that SPIN1 is indeed a positive regulator of the MDM2‐p21‐E2F1 signaling pathway. Interestingly, E2F1 could in turn directly bind to the SPIN1 promoter region and activate its expression at the mRNA and protein levels, thus forming a positive feedback loop that drives the malignant behavior of GC.

As expected, only p21 protein expression decreased and increased when SPIN1 was overexpressed and knocked down, whereas p21 mRNA levels were not affected. The results were concordant with earlier reports that MDM2 mediates p21 proteasomal degradation [28, 30]. We next demonstrated that SPIN1 regulates the expression of MDM2 by binding to H3K4me3. As a histone code reader, SPIN1 has been reported to recognize H3K4 methylation and stimulate the expression of rRNA genes [18]. Our results complemented the role of SPIN1's reader function in the development of tumorigenesis. Besides, as MDM2 is an important target gene for p53, we wonder to know whether p53 is involved in the regulation of MDM2 by SPIN1, and then, rescue experiments were performed. The results indicated that regulation of MDM2 by SPIN1 is independent of p53, which also validates the direct regulation of MDM2 by SPIN1.

To further explore whether SPIN1 can be used as a target for GC therapy *in vivo*, we constructed xenograft tumor models. The results demonstrated that lentiviral packaged ShRNA‐SPIN1 could inhibit GC tumor growth, which indicated that SPIN1 might be a promising target for GC therapeutics, and LV‐ShRNA‐SPIN1 gene therapy may be a promising novel approach to treat advanced GC. Our results also provide a reliable theoretical basis for the application of SPIN1 small molecule inhibitors to GC patients.

## 5 Conclusions

In conclusion, we demonstrated for the first time that SPIN1 functions as an oncogene in GC. The elevated expression of SPIN1 and transcriptional activation by E2F1 promotes cell proliferation and cell cycle progression by binding to H3K4me3 of the MDM2 promoter region and activating the MDM2‐p21‐E2F1 pathway. SPIN1 may serve as a potential therapeutic target for the treatment of GC.

## Conflict of interest

The authors declare no conflict of interest.

## Author contributions

BL, HL, and PG conceptualized and designed the manuscript. BL, HL, and PG developed the methodology. BL, HL, XC., RM, GZ, LS., SW, and PG acquired the data. BL, HL, XC., RM, GZ, LS., SW, YW, and PG analyzed and interpreted the data. BL, HL, and PG wrote, reviewed, and/or revised the manuscript.

## Supporting information


**Fig. S1.** The relative expression level of SPIN1 in GC cell lines and regulation of some downstream proteins by SPIN1.
**Fig. S2.** P53 does not play a role in the regulation of MDM2 by SPIN1.
**Table S1.** Expression of SPIN1 protein in nontumorous gastric mucosa, primary gastric cancer tissues and secondary lymph node metastatic foci.
**Table S2.** Univariate and multivariate analysis for overall survival after surgery.
**Table S3.** Several differentially expressed genes in the GEO database under Accession No. GSE71141.

## Data Availability

Microarray data performed by Chen *et al*. [4], on breast cancer cells have been deposited in NCBI Gene Expression Omnibus (GEO), and the Array data are available in the GEO database under Accession No. GSE71141. The raw data of this result are available from the corresponding author upon reasonable request.
