# Supplementary material for: E2F1‐activated SPIN1 promotes tumor growth via a MDM2‐p21‐E2F1 feedback loop in gastric cancer
Source: Mol Oncol. 2020 Aug 26;14(10):2629–45. doi: 10.1002/1878-0261.12778 (PMC7530787; doi:10.1002/1878-0261.12778)
Supplement: Supplementary file 1 — Fig. S1. The relative expression level of SPIN1 in GC cell lines and regulation of some downstream proteins by SPIN1. Fig. S2. P53 does not play a role in the regulation of MDM2 by SPIN1. Table S1. Expression of SPIN1 protein in nontumorous gastric mucosa, primary gastric cancer tissues and secondary lymph node metastatic foci. Table S2. Univariate and multivariate analysis for overall survival after surgery. Table S3. Several differentially expressed genes in the GEO database under Accession No. GSE71141. [file MOL2-14-2629-s001.docx]

**Table S1.Expression of SPIN1 protein in nontumorous gastric mucosa, primary gastric cancer tissues and secondary lymph node metastatic foci**

| **Tissue samples** | N | **SPIN1 expression** | | P value |
| --- | --- | --- | --- | --- |
|  |  | Low （%） | High（%） |  |
| **Nontumorous gastric mucosa** | 59 | 54（91.5） | 5（8.5） | < 0.0001^a^ |
| **Primary gastric cancer tissues** | 113 | 52（46.0） | 61(54.0) | < 0.0001^b^ |
| **Secondary lymph node metastatic foci** | 56 | 17(30.3) | 39(69.6) | 0.0271^c^ |

aDifference between nontumorous gastric mucosa and primary gastric cancer tissues.

bDifference between primary gastric cancer tissues and secondary lymph node metastatic foci.

cDifference between nontumorous gastric mucosa and secondary lymph node metastatic foci.

**Table S2. Univariate and multivariate analysis for overall survival after surgery**

| **Univariate and multivariate analysis for overall survival after surgery** | | | | | | | |
| --- | --- | --- | --- | --- | --- | --- | --- |
| **(Cox proportional hazards regression model)** | | | | | |  |  |
|  |  | Univariate | | analysis | Multivariate | | analysis |
| Variable |  | HR | CI (95%) | P value | HR | CI (95%) | P value |
| Age |  | 0.750 | 0.420-1.337 | 0.329 | 0.567 | 0.286-1.122 | 0.103 |
| Gender |  | 1.173 | 0.583-2.359 | 0.655 | 0.962 | 0.438-2.114 | 0.923 |
| Tumor size | | 0.723 | 0.403-1.297 | 0.277 | 0.781 | 0.407-1.498 | 0.457 |
| SPIN1 express | | 0.195 | 0.091-0.418 | 0.000 | 0.642 | 0.257-1.606 | 0.344 |
| Tumor stage | | 0.173 | 0.077-0.388 | 0.000 | 0.483 | 0.124-1.882 | 0.294 |
| Differentiation | | 0.340 | 0.164-0.705 | 0.004 | 0.486 | 0.184-1.283 | 0.145 |
| Lymph node metasis | | 0.236 | 0.106-0.528 | 0.000 | 1.460 | 0.443-4.809 | 0.534 |
| Distant metasis | | 0.040 | 0.018-0.092 | 0.000 | 0.045 | 0.016-0.125 | 0.000 |

**Table S3. Several differentially expressed genes in the GEO database under Accession No. GSE71141.**

| **SEQ_ID** | **Fold change(Si] vs [NC]** | **Log Fold change** | **Absolute Fold change** | **Regulation** | **GENE_NAME** | **SYNONYMS** | **DESCRIPTION** | **NCBI_GENE_ID** |
| --- | --- | --- | --- | --- | --- | --- | --- | --- |
| BC032783 | -11.489051 | -3.5221877 | 11.489051 | down | GPNMB | HGFIN\|NMB | glycoprotein (transmembrane) nmb | 10457 |
| BC036660 | -4.741419 | -2.245319 | 4.741419 | down | MAPKBP1 | - | mitogen activated protein kinase binding protein 1 | 23005 |
| NM_006880 | -4.0960073 | -2.0342183 | 4.0960073 | down | MDM2 | MGC71221\|hdm2 | Mdm2, transformed 3T3 cell double minute 2, p53 binding protein (mouse) | 4193 |
| U89330 | -3.8709464 | -1.9526863 | 3.8709464 | down | MAP2 | DKFZp686I2148\|MAP2A\|MAP2B\|MAP2C\| | microtubule-associated protein 2 | 4133 |

**
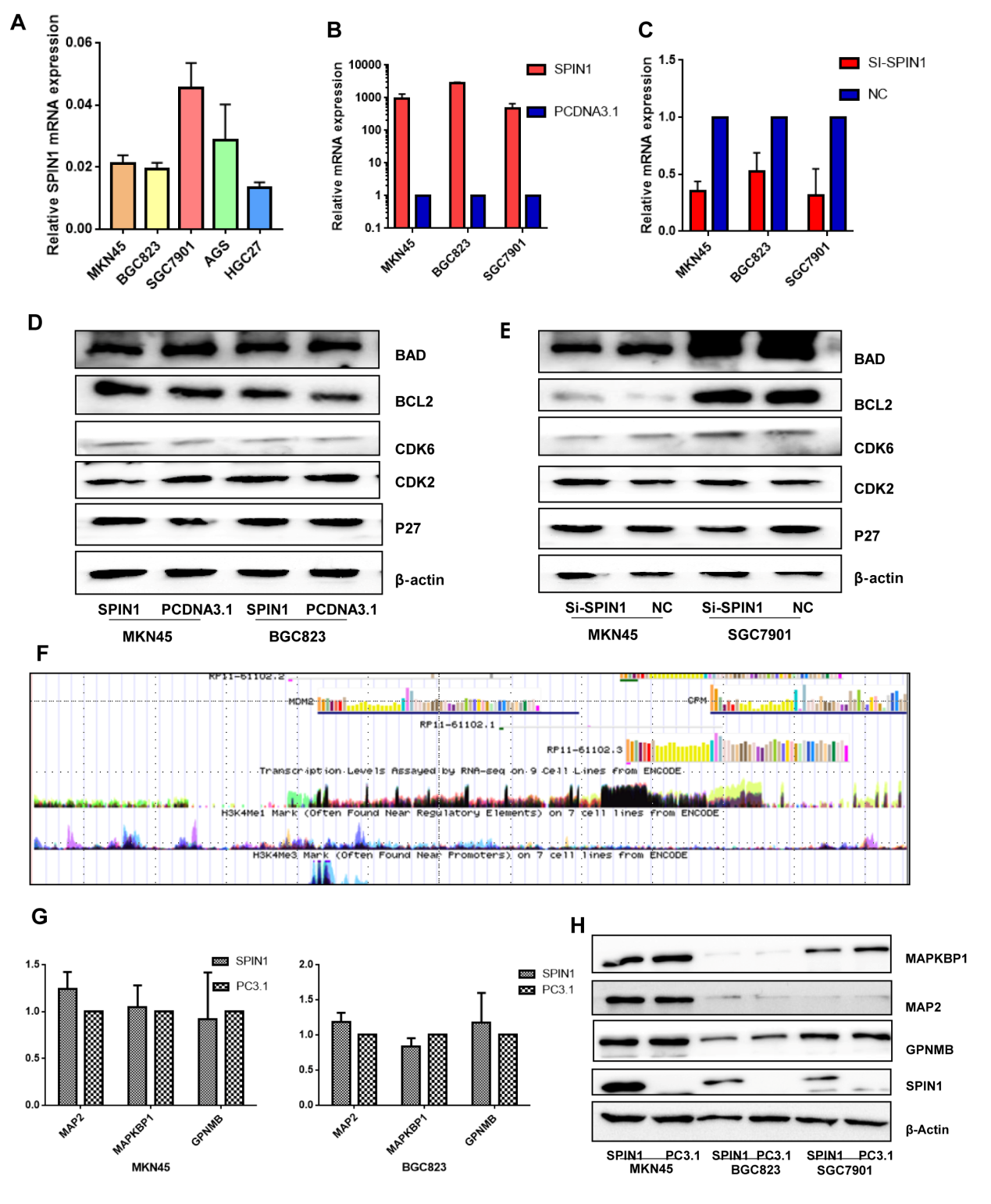
**

**Fig. S1.** **The relative expression level of SPIN1 in GC cell lines and regulation of some downstream proteins by SPIN1.** (A) The relative expression level of SPIN1 in five GC cell lines. SPIN1 expression was highest in poorly differentiated and metastatic cell line SGC 7901. Three independent experiments were performed, and data are presented as mean ± SD. (B, C) Q-PCR showed overexpression or interference of SPIN1 can significantly increase or decrease the mRNA level of SPIN1. Three independent experiments were performed, and data are presented as mean ± SD. (D-E) Western blot results showed that there was no significant difference in the protein expression of p27, CDK2, CDK6, BCL2 and BAD when SPIN1 was overexpressed or knocked down. Three independent experiments were performed. (F) UCSC was used to predict upstream regulation of MDM2. H3K4me1, H3K4me3, and H3K27AC marks were found in seven cell lines from ENCODE. (G) No significant effects in mRNA levels of MAP2, MAPKBP1, GPNMB were observed when SPIN1 is overexpressed in MKN45 and BGC823. Three independent experiments were performed, and data are presented as mean ± SD. (H) No significant effects in protein expression of MAP2, MAPKBP1 and GPNMB were observed when SPIN1 is overexpressed in three GC cell lines. Three independent experiments were performed.


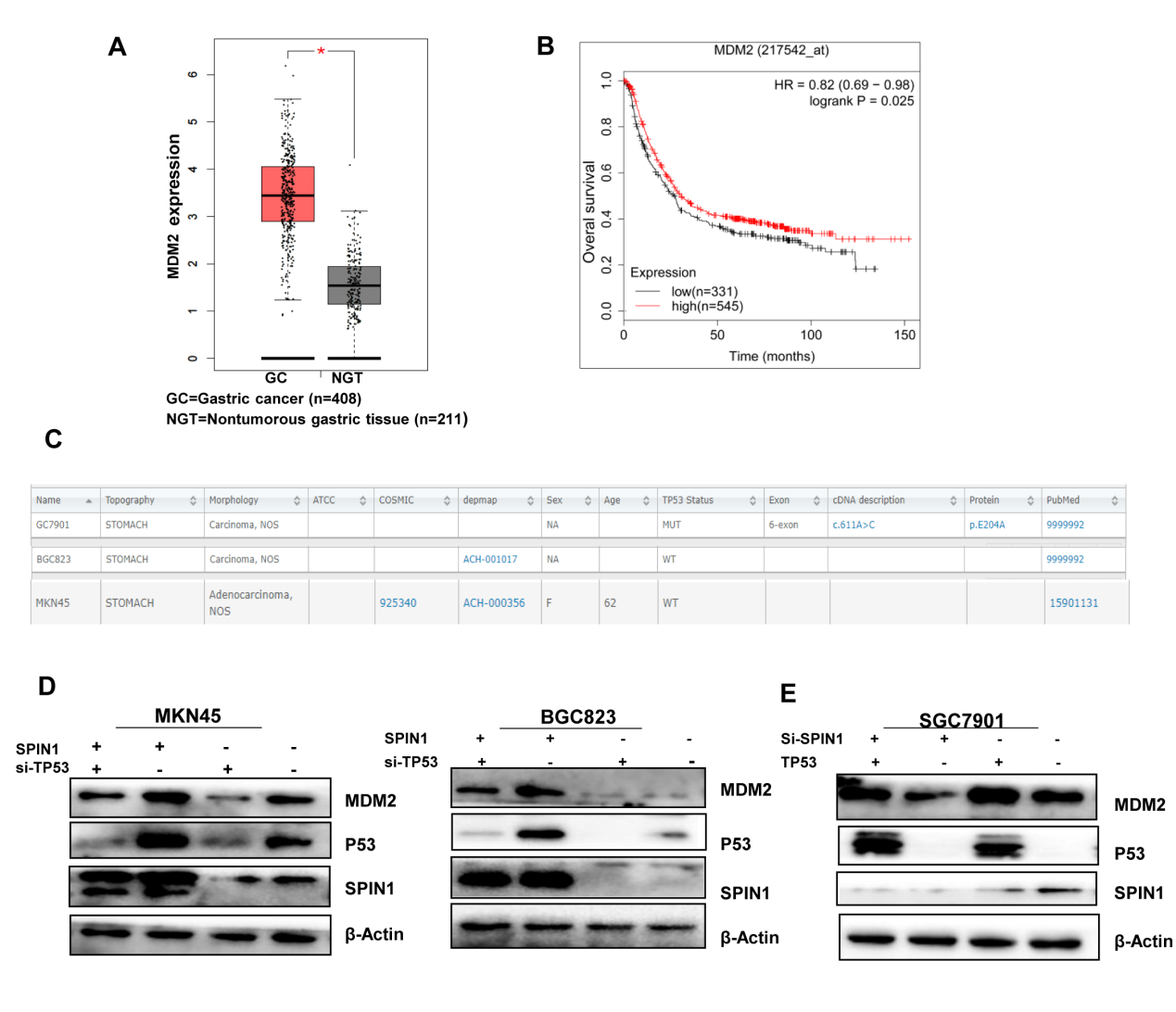


**Fig. S2.** **P53 does not play a role in the regulation of MDM2 by SPIN1.** (A) MDM2 expression is markedly elevated in human gastric cancer samples compared with normal tissues from the TCGA databases using GEPIA（n = 619, t-test, *P* < 0.05）. Data are presented as mean ± SD. (B) The Kaplan-Meier Plotter showed that patients with high expression of MDM2 had poorer overall survival (n = 876, logrank *P* = 0.025). (C) IARC TP53 database was used to specify the status of TP53 in three gastric cancer cell lines. The results indicated that MKN45 and BGC823 are wild type TP53 cell lines, while SGC7901 is mutant. (D) The results of western blotting showed that when P53 is knockdown, SPIN1 overexpression still increased the protein expression of MDM2 in MKN45 and BGC823 (wile type TP53) cells. Three independent experiments were performed. (E) When wild type TP53 is overexpressed, SPIN1 knockdown still decreased the protein expression of MDM2 in SGC7901 (mutant TP53) cell. Three independent experiments were performed.
